# A Right Ventricular Mass in a Patient with Squamous Cell Lung Cancer: A Case Report and Review of Literature

**DOI:** 10.7759/cureus.2261

**Published:** 2018-03-02

**Authors:** Lina Ya'qoub, Katie Payne, Shailja Parikh, Jonathan Enriquez

**Affiliations:** 1 Internal Medicine, University of Missouri - Kansas City; 2 Medical Student, University of Missouri - Kansas City; 3 Cardiology, University of Missouri - Kansas City

**Keywords:** lung cancer, right ventricular mass, cardiac metastasis

## Abstract

Cardiac metastasis is much more common than primary cardiac tumors. Lung cancer is one of the most common primary malignancies to metastasize to the heart. It is not common for metastasis in the heart to present as a cavitary mass. To our knowledge, four cases have been reported in the literature showing metastatic lung cancer to the heart, presenting as a right ventricular mass.

## Introduction

Various types of primary tumors can metastasize to the heart, with lung, breast, melanoma, and lymphoma being the most common sources of metastasis [1-3]. To our knowledge, four cases of cardiac metastasis from lung cancer, presenting as a right ventricular mass, have been reported in the literature. Here, we present a case of squamous cell carcinoma of the lung which was found to have an incidental right ventricular mass on computed tomography (CT) of the chest. The characteristics of the mass, on subsequent echocardiogram, were consistent with a metastatic process.

## Case presentation

A 75-year-old female with a past medical history significant for squamous cell carcinoma of the lung, coronary artery disease, mechanical mitral valve replacement on warfarin, and anemia of chronic disease presented to the emergency room with a two-day history of intractable nausea and vomiting. Her vital signs were within normal limits. Physical exam was unremarkable except for the mechanical click at the cardiac apex, with otherwise normal heart sounds. Her skin was pale as well.

**Figure 1:**
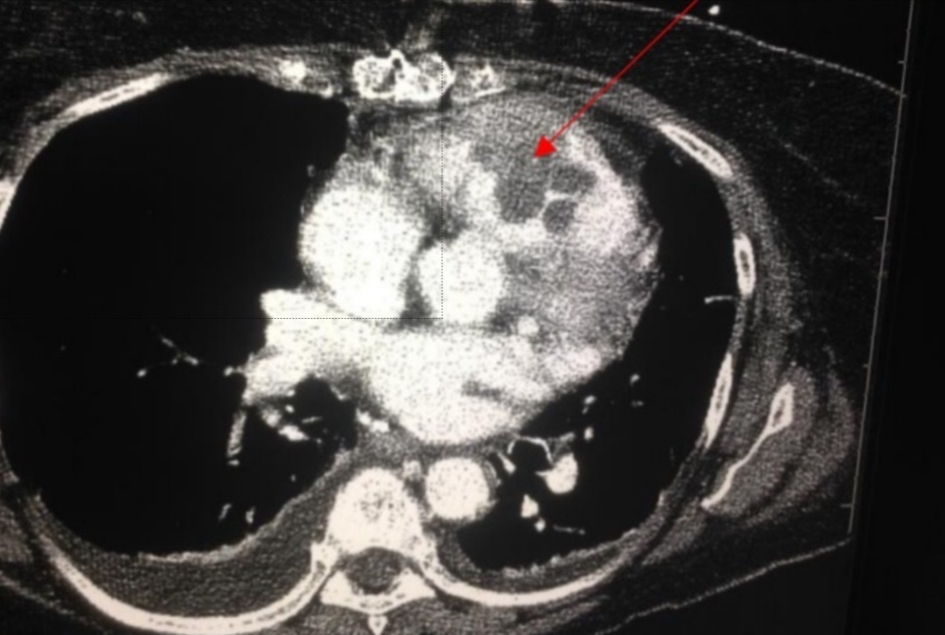
Computed tomography (CT) chest showing a large, multi-lobulated, heterogenous mass in the right ventricle

On hospital day two, her hemoglobin declined from 9.2 g/dL to 6.7 g/dL with no source of overt bleeding. As part of her acute hemoglobin drop, while being on anti-coagulation therapy, CT of the chest, abdomen, and pelvis was obtained to look for occult bleeding. The CT scan did not reveal a source of bleeding, but it showed an increase in the size of the left upper lobe lung mass, worsening of the associated central necrosis of the mass, as well as a new multi-lobulated mass in the right ventricle and right ventricular outflow tract (Figure 1). Further imaging with an echocardiogram was recommended.

**Figure 2:**
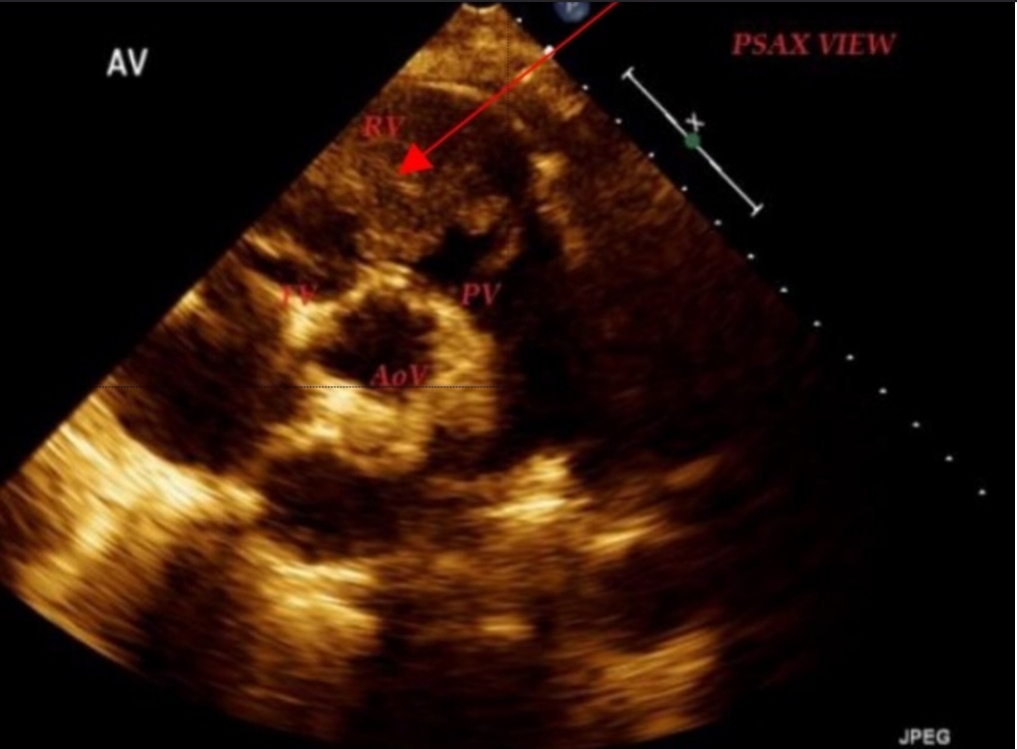
Parasternal short axis view echocardiographic image showing a large fungating mass in the right ventricle RV: right ventricle; PV: pulmonary valve; AoV: aortic valve; TV: tricuspid valve.

The echocardiogram showed a large 4.0 cm x 3.5 cm fungating heterogenous mass originating from the free wall of the right ventricle and prolapsing through the pulmonic valve (Figure 2). Given the mass location, and the characteristic features and history of squamous cell lung carcinoma, the mass was deemed to be likely metastatic in origin by the reading of two board-certified cardiologists.

The patient and family declined further evaluation and preferred hospice care. The patient passed away six weeks after discharge.

## Discussion

Cardiac metastases are much more common than primary cardiac tumors, with lung, breast cancers, lymphomas, and melanomas being the most common malignancies to metastasize to the heart [1]. Mukai et al. reported that only one case of primary tumor (malignant mesothelioma) was identified among 2649 autopsies of malignant tumors at the National Cancer Center Hospital [2]. Although the vast majority of the cases of cardiac metastases are asymptomatic and thus diagnosed at autopsy, patients may have various clinical presentations depending on the anatomical cardiac compartment involved as well as the tumor burden. Patients may present with chest pain, dyspnea, palpitations, arrhythmias, pericardial effusion, or tamponade [3-6].

The pericardium is the most common site involved in approximately 70%-80% of all cases of cardiac metastases. The epicardium and myocardium involvement is less common with up to 10%-15% of cases, and the endocardium involvement is the least common [5]. While pericardium and epicardium involvement is usually a result of direct contiguous extension or lymphatic involvement, myocardium involvement usually indicates hematogenous spread. Endocardium involvement is a result of hematogenous spread or extension from the myocardium [4,6-7].

Cardiac metastasis from lung cancer presenting as a cavitary mass is rare. To our knowledge, there are four case reports of lung cancer with right ventricular involvement presenting as a mass on imaging. These cases are summarized in Table 1.

**Table 1:** Table of case reports of right ventricular metastasis from lung cancer presenting as a cavitary mass RV: right ventricle; PET: positron emission tomography; FDG: fluoro-deoxy glucose; CT: computed tomography; MRI: magnetic resonance imaging.

Author	Location of mass	Imaging modality	Biopsy
Kim et al. [3]	RV wall	Echocardiogram, CT, MRI, FDG-PET	Not done
Shiose et al. [5]	RV free wall	PET	Mass was resected
Orcurto et al. [6]	RV myocardium	FDG-PET, CT	Done
Shah et al. [7]	RV	Echocardiogram, CT	Not done

Although the definitive diagnosis is made by biopsy, multi-modality imaging techniques including echocardiography, CT, magnetic resonance imaging (MRI), fluoro-deoxy glucose positron emission tomography (FDG PET), are useful and widely-used diagnostic tools. Furthermore, the characteristics of the lesions in some cases, in the setting of malignancy history, may preclude the need for biopsy for diagnostic purposes [5,8]. Bruce and colleagues have previously reported the utility of these imaging techniques and the characteristics of cardiac metastases on imaging, including heterogeneity of the mass, size, multi-lobulated shape, and enhancement with contrast [1,8].

Management of cardiac metastases varies depending on the patient symptoms, burden of the tumor, prognosis, and the patient’s preferences [6]. While surgery is an option for patients with isolated cardiac metastasis, most cases of cardiac metastases are associated with multiple metastases elsewhere in the body. Chemotherapy, radiotherapy, or palliative treatment is often offered to those with multiple metastatic sites and who are not candidates for surgery [1,6]. 

## Conclusions

In conclusion, cardiac metastases are much more common than primary cardiac tumors. Physicians should keep cardiac metastasis in their differential diagnosis when they evaluate patients with cardiac masses, especially in those with a known history of malignancy. Echocardiography and multi-modality imaging are useful and commonly-used techniques to approach and diagnose cardiac masses.
